# Acute suppurative thyroiditis due to *Streptococcus anginosus* leading to sepsis and acute respiratory distress syndrome: a case report

**DOI:** 10.20945/2359-3997000000420

**Published:** 2021-11-11

**Authors:** Christian Trummer, Verena Theiler-Schwetz, Eva Steinberger, Alexander C. Reisinger, Eva Hassler, Thomas Valentin, Sabine Reinisch, Stefan Pilz

**Affiliations:** 1 Medical University of Graz Department of Internal Medicine Division of Endocrinology and Diabetology Graz Austria Division of Endocrinology and Diabetology, Department of Internal Medicine, Medical University of Graz, Graz, Austria; 2 Medical University of Graz Department of Internal Medicine Intensive Care Unit Graz Austria Intensive Care Unit, Department of Internal Medicine, Medical University of Graz, Graz, Austria; 3 Medical University of Graz Department of Radiology Division of Neuroradiology, Vascular and Interventional Radiology Graz Austria Division of Neuroradiology, Vascular and Interventional Radiology, Department of Radiology, Medical University of Graz, Graz, Austria; 4 Medical University of Graz Department of Internal Medicine Section of Infectious Diseases and Tropical Medicine Graz Austria Section of Infectious Diseases and Tropical Medicine, Department of Internal Medicine, Medical University of Graz, Graz, Austria; 5 Medical University of Graz Department of Otorhinolaryngology Division of General Otorhinolaryngology, Head and Neck Surgery Graz Austria Division of General Otorhinolaryngology, Head and Neck Surgery, Department of Otorhinolaryngology, Medical University of Graz, Graz, Austria

## Abstract

Acute suppurative thyroiditis (AST) is a rare but potentially life-threatening thyroid disease with a high mortality if left untreated. Thus, differentiation from other thyroid disorders is highly important in clinical practice. A 22-year-old male patient was admitted to a tertiary care hospital with cervical pain, palpitations, thyrotoxicosis, and an inhomogeneously enlarged right thyroid lobe. In view of the clinical findings, subacute thyroiditis (SAT) was suspected and treatment with glucocorticoids was started. After initial amelioration, the patient developed cervical erythema, fever, and recurrent pain. A CT scan showed extensive phlegmonous inflammation and abscess formation, suggestive of AST. We started immediate empiric antibiotic therapy and performed surgical drainage of the abscess formations. Subsequently, the patient developed hypoxic respiratory failure, leading to ICU admission and intermittent need for non-invasive ventilation. Blood and abscess cultures were positive for *Streptococcus anginosus*. If left untreated, AST represents a potentially life-threatening disease. Thus, in clinically doubtful cases, liberal further assessment by means of cervical CT scans or fine needle aspiration biopsy are strongly advised.

## INTRODUCTION

A cute suppurative thyroiditis (AST) is a rare but potentially lethal thyroid disease (1). It has an estimated prevalence of 0.1-0.7 percent of all thyroid disorders affecting women and men at an even proportion (2,3). AST is an acute infection of the thyroid gland. In the vast majority of cases, causative agents are bacterial pathogens (1). The risk of infection is significantly increased by pre-existing anatomic or structural variants, e.g. piriform sinus fistulas, which can be found in more than 70 percent of AST cases (4). Patients usually present with neck swelling, localized pain and erythema, fever, hoarseness, and dysphagia (1,4). Characteristic biochemical findings include elevated inflammation parameters such as C-reactive protein (CRP) and increased serum thyroglobulin as a marker for destructive thyroiditis (1,5). In contrast to other causes of destructive thyroiditis, e.g. subacute thyroiditis (SAT) or postpartum thyroiditis, thyrotoxicosis is uncommon and occurs in less than 20 percent of patients with AST (4). In an acute setting, thyroid sonography and computed tomography (CT) are generally preferred as imaging studies (6). Due to the potentially high mortality, immediate empiric antibiotic therapy is mandatory in all cases of AST. Additionally, blood cultures, abscess fluid or tissue samples should be obtained (1). Surgical drainage or subtotal/total thyroidectomy is necessary in severe cases of AST or in case of deterioration despite antimicrobial therapy and/or minimal-invasive drainage (1,7).

Due to its rarity, the diagnosis of AST can be challenging. However, in view of its potentially dramatic or even lethal course, differentiation from other thyroid disorders with higher prevalence such as SAT is of utmost clinical importance (1). In this report, we describe the case of a 22-year-old man with AST initially treated with glucocorticoids for suspected SAT. The patient eventually developed sepsis with acute respiratory distress syndrome (ARDS) due to *Streptococcus anginosus* bacteremia. We discuss current recommendations for diagnosis and treatment of AST with particular focus on the differentiation from other thyroid disorders.

## CASE PRESENTATION

A 22-year-old male patient was admitted to the otorhinolaryngology (ENT) outpatient clinic of a tertiary care hospital in October 2019. The patient complained of cervical supraclavicular and prelaryngeal pain lasting for two weeks aggravated by swallowing as well as increased sweating and palpitations. The patient had experienced symptoms of an upper respiratory tract infection prior to the onset of the cervical discomfort. While the inspection of nose, larynx and pharynx in the ENT department was inconspicuous, the right thyroid lobe was prominent on palpation and inhomogeneously enlarged in sonography. Therefore, the patient was referred to the endocrine outpatient clinic with suspected thyroiditis.

**Figure 1:**
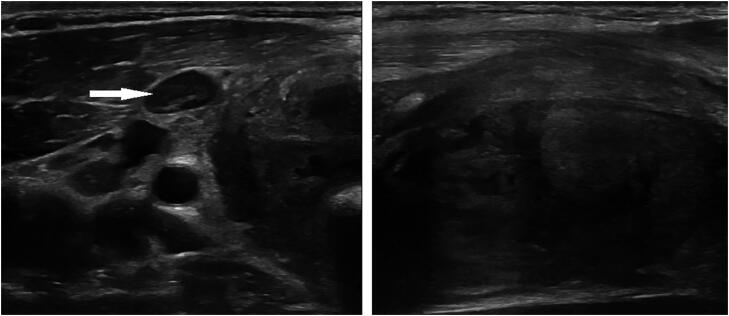
Sonography of the right thyroid lobe at initial presentation (left image: transverse axis, right image: sagittal axis, arrow: prominent lymph node). Images in this figure were previously published in (33) under the Creative Commons CC BY license.

**Figure 2:**
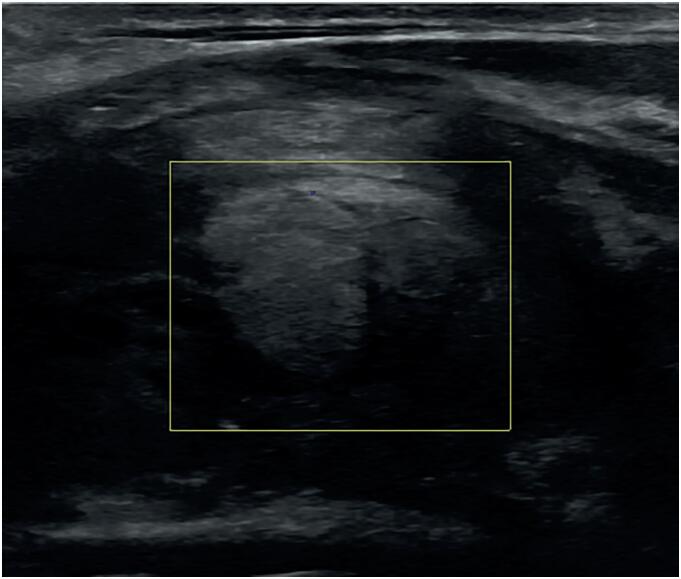
Color-coded Doppler sonography of the right thyroid lobe at initial presentation (sagittal axis).

At the time of admission, the patient did not receive any specific medical therapy; however, he reported regular anabolic androgenic steroid (AAS) abuse for muscle gain. Thyroid function tests showed thyrotoxicosis (TSH: 0.04 µU/mL, reference range 0.10-4.00, fT4: 30.7 pmol/L, reference range 9.5-24.0, fT3: 8.7 pmol/L, reference range 3.0-6.3). Thyroid-specific antibodies (thyroperoxidase antibodies, thyroglobulin antibodies, TSH-receptor antibodies) were not elevated. As a marker for destructive thyroiditis, the serum thyroglobulin concentration was significantly elevated (231 ng/mL, reference range 0-30). The patient was afebrile, but inflammation parameters were moderately increased (white blood count: 11.42/µL, reference range 4.4-11.3, CRP: 38.5 mg/L, reference range 0-5), liver parameters (including aspartate aminotransferase and alanine aminotransferase) were within the normal range. Detailed sonography revealed a significantly enlarged right thyroid lobe (2.6 x 2.8 x 5.8 cm in diameter) with markedly inhomogeneous structure, reduced echogenicity and decreased vascularization in color-coded Doppler imaging (Figure 1, Figure 2). Furthermore, a prominent oval-shaped lymph node with a longitudinal diameter of 1.11 cm was found adjacent to the right interior jugular vein (Figure 1). In contrast to these findings, the left thyroid lobe showed normal size and echogenicity as well as a homogenous structure. There were no cystic or nodular thyroid lesions.

**Figure 3:**
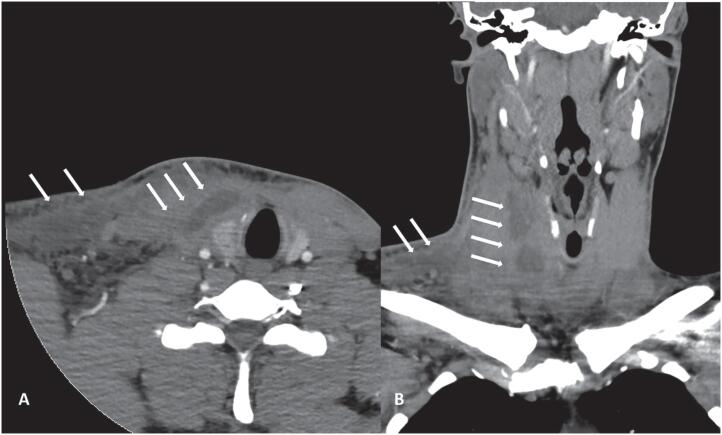
Contrast-enhanced computed tomography of the neck, showing large confluent central hypodense contrast formation (arrows) starting from the right thyroid lobe and extending to the subcutis ventral of the sternocleidomastoid muscle in axial (**A**) and frontal (**B**) orientation.

In view of the clinical and biochemical findings with thyrotoxicosis, affection of the left thyroid lobe, and the absence of fever (which are all uncommon in AST), SAT was suspected and treatment with 50 mg of prednisolone once daily and 10 mg of propranolol three times per day was started. We discussed the main differential diagnosis between SAT and AST with the patient and instructed him to immediately return to the outpatient clinic in case of lacking clinical improvement within the next few days. Following initiation of glucocorticoid and beta-blocker treatment, the patient reported rapid and significant amelioration of pain and symptoms of thyrotoxicosis in a phone call with the treating physician. However, one week later, the patient noticed recurrence of cervical pain and localized erythema extending from the right thyroid lobe to the pectoral region and fever, and presented at the medical emergency department. Compared to the initial presentation, inflammation parameters had significantly increased (white blood count: 36.64/µL, CRP: 192.3 mg/L, procalcitonin: 2.98 ng/mL, reference range: 0-0.5). A CT scan of the neck and thorax showed a phlegmonous process of 8 x 4 x 9 cm in diameter with abscess formation, reaching from the hyoid to the infraclavicular space with compression of the right internal jugular vein (Figure 3). After collection of blood cultures and initiation of parenteral antibiotic treatment with piperacillin/tazobactam, the patient was referred to the ENT department for surgical intervention. During the first postoperative days following successful surgical drainage of several abscesses, the patient developed progressive dyspnea. Due to hypoxemic respiratory failure, the patient was referred to the medical intensive care unit (ICU). CT showed bilateral inflammatory pulmonary infiltrates, concordant with moderate ARDS (paO_2_/FiO_2_ ratio 152). No signs of heart failure or endocarditis were present in echocardiography. Blood and abscess fluid cultures were positive for *Streptococcus anginosus*. The patient initially required non-invasive ventilation support. Treating ICU physicians continued parenteral antimicrobial treatment with piperacillin/tazobactam and added moxifloxacin. In addition, methylprednisolone was administered and regular lavages of the cervical drainages were performed. Inflammation parameters decreased and the patient’s clinical status improved. The patient was discharged from ICU care after 10 days and from inpatient care after a total of 19 days.

Subsequent routine outpatient visits showed a normalization of thyroid function parameters; inflammation parameters remained within the normal range. In sonography, both thyroid lobes were of normal size with homogeneous parenchyma and without any nodal or cystic lesions or adjacent enlarged cervical lymph nodes. There was no evidence of anatomic or cervical structural variants that may represent a predisposition for developing AST.

## DISCUSSION

In this report, we present a rare case of AST that lead to *Streptococcus anginosus* sepsis with ARDS requiring ICU treatment. Our report underlines the importance of AST as a possible life-threatening disease, especially in view of the patient’s young age and lack of relevant medical history.

A distinction of AST from other thyroid disorders, in particular SAT, is of utmost importance in the diagnostic process (1). SAT represents the most common granulomatous thyroid disease with a probable viral or post-viral etiology and an incidence of approximately 12.1 cases per 100.000 individuals per year (8-10). In comparison to AST, thyrotoxicosis as a consequence of destructive thyroiditis is a common finding in SAT (9). Furthermore, abnormal liver function tests, including elevated concentrations of transaminases, alkaline phosphatase, and gamma-glutamyl transpeptidase, are occasionally found in SAT patients (11). Thyroid sonography may be helpful in establishing a diagnosis, as AST is usually found unilaterally (mainly in the left thyroid lobe), while SAT commonly affects both thyroid lobes (6,12). However, in the early inflammatory stage of AST, abscess formation is virtually always absent in sonography, while a unilateral hypoechoic area is frequently found (6). Thus, SAT is a common misdiagnosis in the acute setting (6). This was also the case in our patient, who showed no clear abscess structure in thyroid ultrasound at first presentation. The initial diagnosis of SAT was further supported by the presence of thyrotoxicosis, whereas liver function tests were normal. Additionally, like in our patient, the rapid and significant amelioration of symptoms under glucocorticoid treatment – while characteristic for SAT – may also occur transiently in AST, thus leading to a possible misdiagnosis (13). As it may be tempting to omit invasive procedures after achieving an excellent clinical response under glucocorticoids, we would like to emphasize that liberal fine needle aspiration biopsy should be performed in all doubtful cases, especially if diagnostic uncertainty remains after sonographic imaging (1,14,15). Though very rarely observed, AST must also be differentiated from aggressive thyroid carcinomas (e.g., anaplastic or medullary thyroid carcinomas), as these entities may also cause local infections and necrosis (1).

*Streptococcus anginosus* is not an uncommon pathogen in AST, as approximately 39% of all cases are attributed to gram-positive aerobes (1-3,16). It belongs to the *Streptococcus milleri* group, a family of three genetically distinct microorganisms characteristically involved in pyogenic processes in humans (17). In particular, a distinct association of *Streptococcus anginosus* with AST was described in previous cases (18,19). All reported patients were treated with parenteral antibiotics and received interventional drainage (18,19). In our patient, empiric antibiotic therapy with piperacillin/tazobactam was initiated after AST was suspected clinically. Antibiotic susceptibility testing from blood and abscess fluid cultures found the detected strain susceptible to the chosen agent. Later in the course, treated ICU physicians added moxifloxacin to the antibiotic regimen due to distinct persistent bilateral pneumonic infiltrates and the need for respiratory support.

Even though the exact morbidity and mortality of AST is currently unknown due to its low incidence, it undoubtedly remains a potentially life-threatening disease with increased mortality in the absence of immediate intervention (1). While almost all of the previously reported patients required parenteral antibiotic treatment, surgical drainage and inpatient care, our case showed a particularly severe course with sepsis and ARDS leading to ICU admission (4,20,21). The reported regular abuse of parenteral AAS presents a potential risk for aggravated forms of AST: aside from the risk of local infections and abscess formation due to intramuscular application, systemic effects of AAS may adversely affect the course of infectious diseases (22). In general, study data suggests an immunosuppressive effect of male sex hormones, leading to an increased susceptibility for bacterial and viral infections (23-25). This may be explained by reduced antibody concentrations in plasma under testosterone influence (26,27). Further, testosterone also decreases pro-inflammatory lymphokine production and increases anti-inflammatory lymphokine secretion (28-30). Thus, regular parenteral injections may also play a role in the etiology of AST in our case, especially since there was no predisposing anatomic or immunologic condition. Notably, cases of AST have been also reported in intravenous drug users (31,32).

Our case report stands out due to the rarity of AST as well as the particularly difficult differentiation from SAT that has been suggested by clinical features at first presentation. Furthermore, we present a case of AST with a serious course in an otherwise healthy young patient, highlighting the potential threat posed by this thyroid disease. Limitations of our case report include the lack of long-term follow-up data as well as the lack of the patient’s perspective on the received treatment. Moreover, we did not assess the erythrocyte sedimentation rate (ESR) in this particular patient, although this would have been indicated in our opinion. Therefore, we cannot rule out that measurement of the ESR would have been helpful in distinguishing between AST and SAT in our patient.

In conclusion, AST represents a rare but potentially life-threatening bacterial thyroid infection that requires immediate medical attention, empiric antibiotic therapy and interventional abscess drainage. However, differentiation from other thyroid disorders, especially SAT, can be difficult as the absence of fever as well as prompt clinical improvement in response to glucocorticoid treatment can also be observed in AST, as illustrated by our case. This is especially important in view of the probably significant mortality associated with unrecognized and thus untreated AST. Therefore, in doubtful cases, liberal additional diagnostic measures such as CT scans of the neck region or fine needle aspiration biopsy are strongly advised. In severe cases of AST, surgical intervention and drainage are necessary.
